# Immediate Effect of Cryo-Compression Therapy on Biomechanical Properties and Perfusion of Forearm Muscles in Mixed Martial Arts Fighters

**DOI:** 10.3390/jcm13041177

**Published:** 2024-02-19

**Authors:** Robert Trybulski, Adrian Kużdżał, Marta Bichowska-Pawęska, Andriy Vovkanych, Adam Kawczyński, Grzegorz Biolik, Jarosław Muracki

**Affiliations:** 1 Medical Department Wojciech Korfanty Upper Silesian Academy, 40-659 Katowice, Poland; 2 Institute of Health Sciences, College of Medical Sciences, University of Rzeszów, 35-959 Rzeszów, Poland; kuzdzal.a@gmail.com; 3 Faculty of Physical Education, Gdansk University of Physical Education and Sport, 80-336 Gdansk, Poland; marta.bichowska@awf.gda.pl; 4 Department of Physical Therapy and Ergotherapy, Ivan Boberkyj Lviv State University of Physical Culture, 79007 Lviv, Ukraine; avovkinfiz@i.ua; 5 Department of Biomechanics and Sport Engineering, Gdansk University of Physical Education and Sport, 80-336 Gdansk, Poland; kawczynski.a@gmail.com; 6 Department of General Surgery, Vascular Surgery, Angiology and Phlebology, Medical University of Silesia, 40-635 Katowice, Poland; gbiolik@sum.edu.pl; 7 Institute of Physical Culture Sciences, Department of Physical Education and Health, University of Szczecin, 70-453 Szczecin, Poland; jaroslaw.muracki@usz.edu.pl

**Keywords:** cryo-compression, microcirculation, myotonometry, game ready, sports recovery, MMA, forearm muscles, fighters

## Abstract

Mixed martial arts (MMA) fighters use their arms and hands for striking with the fists, grappling, and defensive techniques, which puts a high load on the forearms and hand muscles. New methods are needed to decrease the risk of injury and increase the effectiveness of regeneration. This study aimed to assess the effectiveness of cryo-compression (CC) therapy of different times (3 and 6 min) on forearm muscles in MMA fighters by investigating muscle pain, stiffness, tension, elasticity strength, and perfusion. Twenty professional male MMA fighters aged 26.5 ± 4.5 years, with training experience of 10.3 ± 5.0 years, were enrolled on an experimental within-group study design. The participants underwent CC therapy at a temperature of 3 °C and compression of 75 mmHg for 3 min and, in the second session, for 6 min. The investigated parameters were in the following order: (1) perfusion in non-reference units (PU), (2) muscle tone (T—[Hz]), (3) stiffness (S—[N/m]), (4) elasticity (E—[arb]), (5) pressure pain threshold (PPT—[N/cm]), and (6) maximum isometric force (Fmax [kgf]) at two time points: (1) at rest—2 min before CC therapy (pre) and (2) 2 min after CC therapy (post). There were significant differences between 3 and 6 min of CC therapy for PU and T. Meanwhile, F, E, PPT, and S were significantly different when comparing pre- to post-conditions. These results provide evidence that CC therapy is a stimulus that significantly affects parameters characterizing muscle biomechanical properties, pain threshold, strength, and tissue perfusion.

## 1. Introduction

The nature of mixed martial arts (MMA) fights places high physiological demands on the contestants. MMA is different from other martial arts in that it requires high muscle strength endurance of the forearm muscles in the specific position of the hands protected by special gloves that provide grip and absorb strikes [1]. MMA fighters use fists to punch and grip their opponents. Keeping the fists tight requires isometric muscle contraction, which is much more potent than that in boxing or other combat sports, while grips and submission techniques involve alternate isometric contractions [2]. MMA rules allow for striking and grappling in a standing position and on the ground, and the combat itself involves many explosive actions such as kicking and striking, the effectiveness of which are often critical for the final result of a competition. The energy systems that an MMA fighter activates during a fight are the same as those enabling a repeated sprint effort [3].

Training for combat sports, including MMA, focuses on building athletes’ muscular strength and improving the dynamics of movements. An essential element of training planning is to provide the professional athlete with conditions for muscle regeneration. Excessive muscle tension and stiffness can cause high intramuscular pressure, causing faster muscle fatigue and risk of injury [4]. One of the causes of intramuscular pressure disorders may be a disturbed mechanism of capillary function [5,6].

The popular method used for sports recovery described in the scientific literature is cryo-compression therapy (CC) [7]. Conflicting results have also suggested that cryotherapy could delay and impair the regeneration process. There are no definitive findings about cryotherapy’s effects on muscle regeneration [8,9]. Cryo-compression is aimed at lowering the temperature of the tissues [10,11] and reducing inflammation in the damaged area [12,13,14]. Studies have shown that CC reduces swelling [11,14] and improves tissue perfusion [6,15,16]. However, in terms of perfusion, the evidence is inconclusive as evidence suggests that muscle cooling may not affect skeletal muscle capillary perfusion [17,18]. The authors argue that the lack of observed changes in muscle blood supply means that cooling the skin, which has numerous dermal thermoreceptors, profoundly affects skin vascularity, which helps in thermoregulation, but skeletal muscles do not have thermoreceptors. In addition, the literature shows the analgesic effect of CC [19,20] and also suggests a beneficial effect on post-exercise regeneration, muscle tone, and muscle elasticity [21,22].

As cryotherapy has grown in popularity, clinicians, practitioners, and athletes have sought easy-to-use, quick-to-use, and portable alternatives to cryotherapy. An example of such a therapy is Game Ready, which can be used as local cryotherapy or contrast therapy [23,24]. Game Ready cryo-compression combines the use of a cold stimulus that is applied to a specific tissue in the form of a compression cuff with alternating pressure. We can use a pressure of 15 to 75 mmHg and a temperature of 3 to 45 °C. The procedure duration varies from 10 to 30 min [24,25].

Depending on the type of cold application (ice packs, air cryo-stimulation, nitrogen cryo-stimulation, immersion in water, cold spray, cryo-compression) [12], we can distinguish the different times of application of this stimulus to individual parts of the body for several seconds up to 15 min [13,20,21,24,26]. One of the significant problems is the need for more evidence evaluating the impact of CC time on the biomechanical parameters of muscles, such as muscle tone, stiffness, and elasticity. In this situation, it is crucial to assess short-term changes in biomechanical and viscoelastic properties of muscles using short-term CC. If excessive muscle tension and stiffness can cause high intramuscular pressure, causing faster muscle fatigue and risk of injury, it is worth assessing whether a short time of CC can eliminate these changes. One of the causes of intramuscular pressure disorders may be a disturbed mechanism of capillary function [5]. Schaser et al. [6] observed a significant restoration of diminished functional capillary density, a marked decrease in elevated intramuscular pressure, a reduction in the number of adhering and invading granulocytes, and an attenuation of tissue damage in a rat study after 6 h.

Ultimately, the evidence for the effectiveness of CC use for post-exercise recovery remains anecdotal [24,25]. Hohenauer et al. [26], in a meta-analysis describing various forms of cryotherapy versus non-cooling, passive post-exercise strategies using recovery characteristics after various strenuous exercise protocols up to 96 h, concluded that there is insufficient evidence for the effectiveness of different cryotherapy methods. The authors noted the low number of RTC studies. In particular, there is no evidence for using specific recovery protocols and assessing the impact of the short-term (1–5 min) effects of CC on the biomechanical properties of muscles [26]. The short duration of CC may be important as there is evidence that applying cold for more than 30 min can reduce motor performance by impairing muscle function [27,28].

The main aim of our study was to assess the impact of short CC therapy time (3 and 6 min) on forearm muscles in MMA fighters. Our research hypothesis was that CC therapy could help reduce muscle pain, stiffness, and tension and increase muscle elasticity. The consequence of these changes should be improved muscle strength and perfusion to the examined tissues. Reducing the duration of stimulation through CC can be useful in optimizing recovery processes in sports, especially when we have many contestants and limited equipment availability. This preliminary interventional study was performed to improve the CC therapy intervention and assess its possible benefits depending on the time of application. From the perspective of the conducted research, the connection between muscle perfusion and biomechanical properties of muscles turns out to be innovative. The results of this study will allow for the creation of an appropriate CC time for research into direct muscle recovery.

## 2. Materials and Methods

### 2.1. Participants

Twenty male, healthy MMA (Mixed Martial Arts) fighters (age: 26.5 ± 4.5 years, BMI: 24.75 ± 3.0, training experience: 10.3 ± 5.0) were selected according to the following inclusion criteria: age 18–40, with a minimum of 3 years of training experience in MMA, training at least 4 times a week. All fighters were registered on the tapology.com profile and had at least 3 semi-pro or pro fights. Participants with elevated blood pressure (defined as blood pressure > 140/90 mm Hg), individuals undergoing treatment for injuries or with a history of injury to the forearm or hand, and those with damage or unspecified skin and myofascial lesions of the musculoskeletal system were excluded from the study. Participants were also not allowed to have a tattoo on the measurement site because it interferes with tissue perfusion measurements. Also excluded were cases of extreme fatigue of the subject, fever, infection, or at the expressed request of the subject [24,25]. Study participants had to refrain from training for 24 h and abstain from training for 24 h during the study. Additionally, due to tissue perfusion measurements, participants were asked to refrain from consuming ergogenic beverages for 4 h before the study (the list of ergogenic drinks was given to the participants; only water, milk, and juice were recommended during the study). Exclusion was allowed to occur at any time during the study at a participant’s request. Before participating in the study, the participants read and signed an informed consent to participate in the study. The study was approved by the ethics committee of the National Council of Physiotherapists (No. 9/22 of 6 April 2022) and conducted in accordance with the Declaration of Helsinki.

### 2.2. Study Design

**Figure 1:**
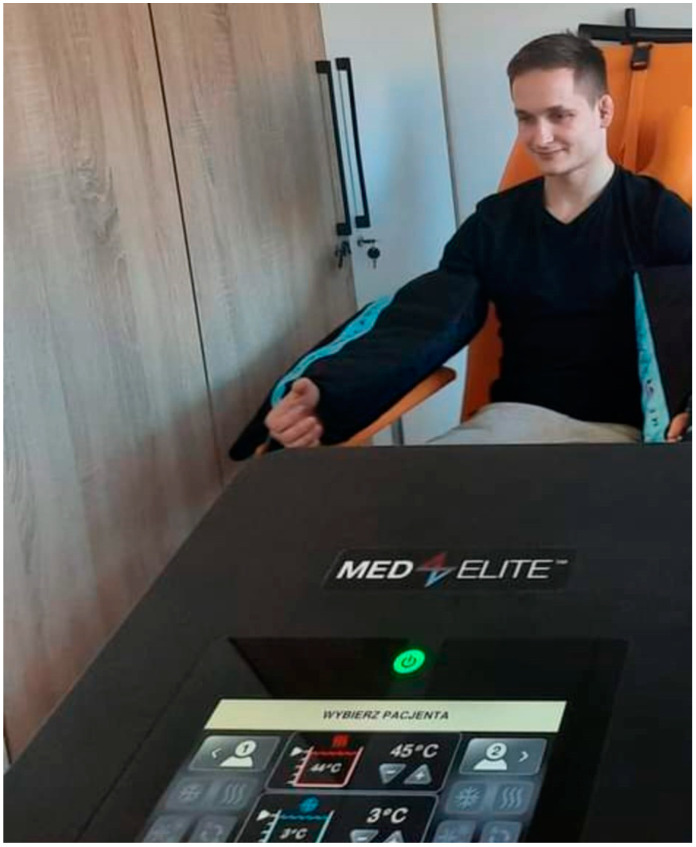
Game Ready contrast therapy equipment. (Authors declare having the participant’s written consent shown in the picture to use it in a scientific article for informative purposes only.)

In this interventional preliminary study, each participant underwent a familiarization intervention before the main phase of the study, which consisted of a 3 min CC with Game Ready equipment stimulation 3 days prior to the study. The experimental intervention used the manufacturer’s Game Ready and clinically shortest cooling dose of 3 min and a second dose of 6 min. The target temperature was manually set at 3 °C and pressure to high compression (75 mmHg) as per the manufacturer’s options with standard, approximately 1 min intermittent cyclical compression of the inflatable cuff on the forearm (Figure 1). After the intervention, data were collected in the same way as at rest (https://gameready.com/, accessed on 1 March 2022).

**Figure 2:**
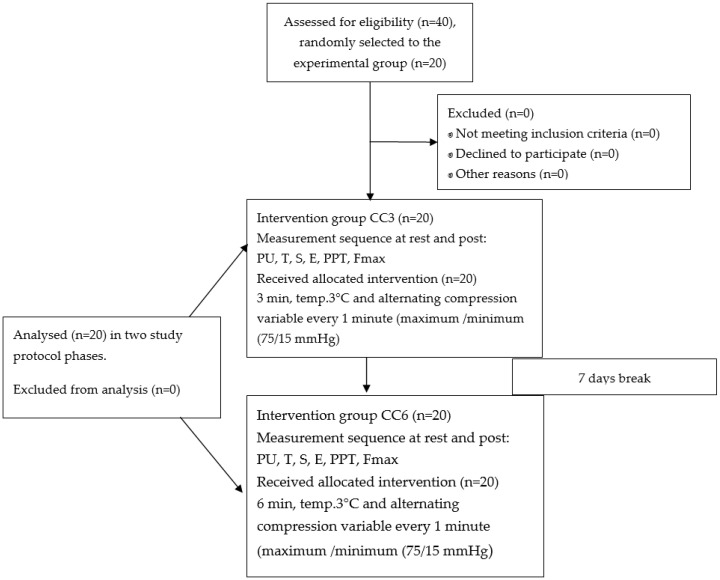
Group allocation and study design.

Two experimental sessions of different CC durations were performed in the same group with a one-week break. The first session was with a stimulation time of 3 min—(CC3), (n = 20) and the second session with 6 min (CC6), (n = 20) (Figure 2). After determining the widest cross-section of the flexor carpi radialis (FCR) muscle of the dominant hand under ultrasound control, the measuring part was marked with a marker [3]. All of the measurements were taken at the same point. The following measurements were taken in all participants of the study: muscle tone (T—[Hz]), stiffness (S—[N/m]), elasticity (E—[arb]), pressure pain threshold (PPT—[N/cm]), microcirculation response described in non-reference units (PU), and maximum isometric force (Fmax—[kgf]). All participants were tested at the same time and under the same conditions (between 10 a.m. and 12 p.m.) in a standardized resting position, sitting in a medical chair with elbows bent at 60° and leaning against the chair [3]. The project was carried out in the Provita Medical Center. All measurements were taken at rest—(pre) 2 min before CGR stimulation and 2 min after CGR—(post). Properly trained physiotherapists performed the measurements. All measurements were taken in the following order: (1) PU, (2) T, (3) S, (4) E, (5) PPT, (6) Fmax, and the procedures are described in detail below.

#### 2.2.1. Measurements—Tissue Perfusion (PU)

The LDF method, due to its repeatability, high sensitivity, and non-invasiveness, allows for a precise assessment of the microcirculation at rest and in response to a physical stimulus. In order to analyze the PU, the Laser Doppler flowmetry (LDF) was used [28]. The wave reflected from the erythrocytes was measured after clinically determining the site [29] and marking it with a special pen [28]. The LDF was performed using a Perimed apparatus (Sweden, 2004). The measurement depth was 2.5 mm, the volume was 1 mm^3^, and the procedure lasted 2 min. To assess augmentation in response to CGR skin microcirculation, the standardized LDF test proposed by Liana et al. [30] was used.

#### 2.2.2. Measurements—Myotonometry

**Figure 3:**
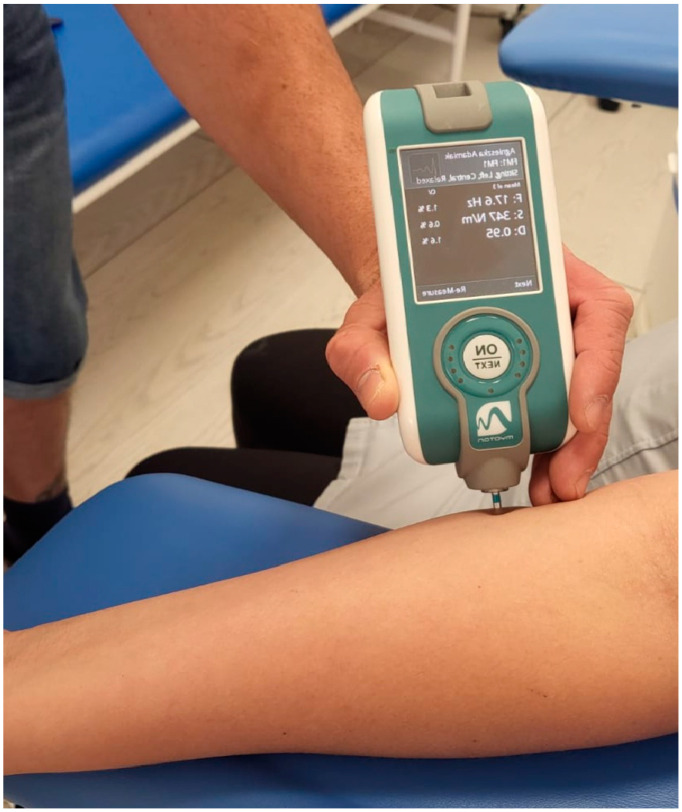
Equipment for myotonometric measurements.

Myotonometry is a reliable measurement method and can detect differences in physical properties of muscles [31,32,33,34], e.g., state of resting muscle tone (T) defined as muted EMG signal and dynamic stiffness (S) [33] and elasticity (E) [35]. The myotonometer test was performed as the second resting and post-stimulation measurement (Figure 3). Measurements were performed using a myotonometer MyotonPRO AS (Myoton Ltd., Tallin, Estonia 2021). Through the probe, a pre-pressure (0.18 N) is applied to the body surface and causes the compression of the material underneath. The device releases a mechanical impulse (0.4 N, 15 ms), deforming the medium for a short interval. Three measurements were taken, and the average values were calculated.

#### 2.2.3. Measurements—Pressure Pain Threshold (PPT)

PPT was the third of all measurements at rest and after stimulation. Pressure pain threshold (PPT) was measured using an algometer FDIX (Wagner Instruments, Greenwich, CT, USA, 2013). Determining the pressure threshold of pain is an attempt to objectively control the so-called resting pain threshold and its changes after therapeutic procedures and physical effort [36]. The subjects were subjected to a three-time compression test with a probe (parameters: r = 4 mm) in a specific tissue area (mm) producing compressive forces. The pressure was applied until the test stimulus was signaled as unpleasant. The force value (kgf) was displayed digitally, calculated as the average of 3 measurements.

#### 2.2.4. Measurements—Maximum Forearm Muscle Strength (Fmax)

Before the test, each subject performed a warm-up consisting of ten times the maximum pressure of a small ball, followed by a 10-s stretching of the forearm muscles. The test was performed using an electronic hand dynamometer Kern MAP 130K1 (Kern, Balingen, Germany). The motion of flexing the fingers of the dominant hand with a 90-degree elbow flexion in a standing position was performed. Each participant had three tries with a 30-s pause in between, and then the average value was calculated.

### 2.3. Statistical Methods

All statistical analyses were performed using Statistica 9.1. Results are presented as means with standard deviations. The Shapiro–Wilk, Levene, and Mauchly’s tests were used in order to verify the normality, homogeneity, and sphericity of the sample data variances, respectively. Differences between the CC3 and CC6 conditions were examined using repeated measures two-way ANOVA (2 conditions × 2 times). Furthermore, *t*-test comparisons for delta values (pre-post) were made between CC3 and CC6 conditions. Effect sizes for main effects and interactions were determined using partial eta squared (η^2^. Partial eta squared values were classified as small (0.01 to 0.059), moderate (0.06 to 0.137), and large (>0.137). Post hoc comparisons using Tukey’s test were conducted to locate the differences between mean values when a main effect or an interaction was found. Percent changes with 95% confidence intervals (95CI) were also calculated. Statistical significance was set at *p* < 0.05. A sample size estimated with an analysis of variance (ANOVA) for within factors, an α of 0.05, a minimum expected effect size (Cohen’s *f*) of 0.25, and β of 0.95 were used. The G*Power software (version 3.1.9.2; Kiel University, Kiel, Germany) [37] was used to estimate the required sample size, setting the aforementioned minimum expected effect size. The procedure returned a minimum number of 12 participants. Finally, accounting for potential dropouts, 20 participants were recruited.

## 3. Results

The Shapiro–Wilk tests indicated that the normality of the data had been violated for PU and T. Therefore, for these parameters to investigate statistical differences, Friedman’s test was used. The Friedman’s test showed significant differences between CC3 and CC6 for PU (Chi-square ANOVA = 31.1; *p* < 0.001; Kendall’s W = 0.52) and for T (Chi-square ANOVA = 14.57; *p* = 0.002; Kendall’s W = 0.25). The pairwise comparisons for PU showed significant differences between rest vs. post values for CC3 (*p* = 0.001; 6.67 ± 2.84 vs. 5.15 ± 2.34, respectively) as well as between rest vs. post values for CC6 (*p* < 0.001; 7.13 ± 3.21 vs. 4.76 ± 1.82, respectively). The pairwise comparisons for T showed significant differences between rest vs. post values for CC3 (*p* = 0.03; 18.58 ± 2.06 vs. 19.90 ± 2.79 [Hz]) as well as for pre- vs. post values for CC6 (*p* = 0.02; 18.19 ± 1.98 vs. 19.66 ± 3.27 [Hz]).

The two-way repeated measures ANOVA did not indicate a significant interaction effect for Fmax (*p* = 0.49; η^2^ = 0.03). However, there was a significant main effect of condition (CC3 vs. CC6; *p* = 0.01; η^2^ = 0.28) as well as for the time of measurement (rest vs. post; *p* < 0.001; η^2^ = 0.73). The post hoc Tukey comparison for the interaction effect showed a significantly higher Fmax for post- compared to premeasurement in CC3 and CC6 (*p* < 0.001 for both; 46.61 ± 9.18 vs. 49.55 ± 8.85 [kgf]; 43.74 ± 8.07 vs. 46.20 ± 8.04 [kgf], respectively). The post hoc Tukey comparison for the main effect showed a significantly higher Fmax for the post- when compared to the premeasurement (*p* < 0.001; 47.9 vs. 45.2 [kgf], respectively). Further, the post hoc Tukey comparison for the main effect of condition (CC3 vs. CC6) showed a significantly higher Fmax for CC3 when compared to CC6 (*p* = 0.01; 48.1 vs. 44.9 [kgf]), respectively).

The two-way repeated measures ANOVA did not indicate a significant interaction effect for S (*p* = 0.18; η^2^ = 0.09). However, there was a significant main effect for time of measurement (rest vs. post; *p* < 0.03; η^2^ = 0.23). The post hoc Tukey comparison for the interaction effect showed a significantly higher S for post- compared to premeasurement in CC3 (*p* = 0.01; 332.5 ± 60.7 vs. 366.2 ± 54.4 [N/m], respectively). The post hoc Tukey comparison for the main effect showed a significantly higher S for post-measurement when compared to premeasurement (*p* = 0.02; 352.8. vs. 328.9 [N/m], respectively).

The two-way repeated measures ANOVA did not indicate a significant interaction effect for E(arb) (*p* = 0.48; η^2^ = 0.02). However, there was a significant main effect for time of measurement (rest vs. post; *p* < 0.001; η^2^ = 0.49). The posthoc Tukey comparison for the interaction effect showed significantly higher E[arb] for post- compared to premeasurement in CC3 (*p* = 0.04; 0.95 ± 0.08 vs. 1.06 ± 0.17 [arb], respectively). The post hoc Tukey comparison for the main effect showed a significantly higher E for post- when compared to premeasurement (*p* < 0.001; 1.03 vs. 0.94 [arb], respectively).

The two-way repeated measures ANOVA did not indicate a significant interaction effect for PPT (*p* = 0.29; η^2^ = 0.05). However, there was a significant main effect for point (pre vs. post; *p* < 0.001; η^2^ = 0.78). The post hoc Tukey comparison for the interaction effect showed significantly higher PPT for post- compared to premeasurement in CC3 and CC6 (*p* < 0.01 for both; 101.1 ± 16.0 vs. 134.6 ± 23.3 [N/cm]; 94.1 ± 18.0 vs. 135.0 ± 21.0 [N/cm], respectively). The post hoc Tukey comparison for the main effect showed a significantly higher PT for post- when compared to premeasurement (*p* < 0.001; 134.8 vs. 97.5 [N/cm], respectively).

**Figure 4:**
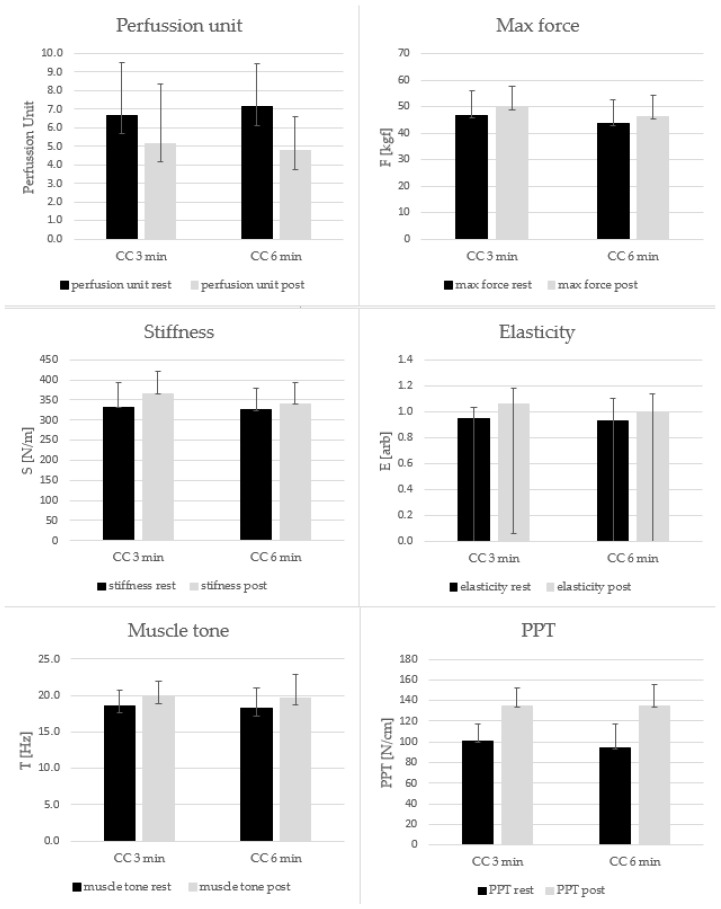
Comparison of analyzed parameters in 3 min vs. 6 min conditions and rest vs. post measurements.

The delta values for PU (post-rest) showed significant differences between CC3 and CC6 conditions (*p* = 0.03; −1.53 ± 2.37 vs. −2.37 ± 2.65, respectively). However, the delta values for PPT did not show significant differences between CC3 and CC6 conditions (*p* = 0.29; 33.5 ± 29.3 vs. 41.0 ± 20.7 [arb], respectively). These results are presented in Table 1 and graphically on Figure 4.

## 4. Discussion

The study’s main objective was to assess the impact of short exposure times to CC on the biomechanical properties of muscles, such as tension, stiffness, and elasticity, as well as the effect on muscle perfusion, pain feeling, and the isometric strength of the forearm. Our results suggest that the effect of 3 min of CC versus 6 min is sufficient and that increasing the time of CC does not increase the effects. There is still debate in the literature about the most effective localized cryo-compression protocol. There are also variations in the recommended “standard” protocol used in sports recovery. Data consistency is not homogeneous [38,39].

### 4.1. Perfusion

The observation of microvascular function has been highlighted to be of clinical importance as it plays a crucial role in the physiological processes of tissue oxygenation and nutritional exchange [16]. Recent evidence suggests that the physiological changes in microcirculation that occur after cryotherapy primarily depend on the reduction in intramuscular temperature [16,40]. Selkov et al. [16] proposed that clinicians should deviate from standard CC treatment times and adjust the cooling time based on subcutaneous tissue thickness. These postulates were consistent with the assumptions of Otte et al. [41], where the authors recommended a specific treatment time based on the thickness of the subcutaneous tissue to achieve a “typical cooling effect”, defined as a reduction in muscle temperature by 7 °C.

Our results suggest that even short-term exposure to cold, even after 3 min, causes the effect of reducing tissue perfusion, which, based on the results of myotonometry, does not cause adverse changes in the biomechanical properties of muscles. It is well known that cold applications initially lead to a strong vasoconstrictive reaction of the skin, contributing to the cold-induced analgesic effect [42]. It has been shown that capillary stenosis in the initial phase of CC can reflexively reduce muscle pain and inflammation [26] and, secondarily, through the expansion mechanism, lead to more efficient regeneration through changes in filtration and reabsorption [8]. This microcirculatory response appears to be used to reduce signs of muscle fatigue [3]. Karunakara et al. [43] analyzed changes in congestion with intermittent CC; however, their study assessed the long-term effect. In the use of CC in two protocols involving 20 min of application and 10 min of break (20/10) and 10 min of application and 10 min of break (10/10), within 1 hour of using this form of cryo-compression, the authors determined that tissue perfusion decreased more intensively with the protocol (20/10) but only after 35 min. Besides the different approaches to assessing the cutaneous microcirculatory properties, the differences in cooling methods might have affected the sports results [42]. Of course, such a short observation time has limitations, and our goal was to check whether the decrease in perfusion disturbs the biomechanical properties of the muscles. Our results showed that changes in perfusion did not negatively affect the biomechanical properties of forearm muscles in MMA fighters.

### 4.2. Biomechanical Properties

Cold and heat stimuli are the subject of numerous studies on the influence on biomechanical properties. However, it should be emphasized that there are no scientific reports in the field of short-term CC. Regarding the biomechanical properties of muscles, we observed significant differences in the increase in muscle tone, elasticity, and dynamic stiffness after CC. During the period of exercise, more than 75% of the energy that is generated by skeletal muscle substrate oxidation is liberated as heat, which inevitably leads to an increase in body temperature [44]. In the situation described, which develops in characteristic atmospheric conditions, additional oxidative stress may occur. Cooling is a practical effect of the application, which is intended to alleviate the effects caused by heat stress and sports performance that may be readily available [45]. Strategies vary widely, and in the case of CGR, there are no clear protocols for determining the timing of changes in hyperemia, stiffness, and essential physical function. The scientific literature confirms that reduced muscle temperature affects contractile power and anaerobic metabolism. So, post-exercise cooling may have regenerative benefits [39].

Bleakley and Costello [46], in a systematic review of the literature, postulated that heat is more effective than cold and should be added to developmental and therapeutic stretching techniques and should be the treatment of choice to enhance the range of motion (ROM) in a clinical or athletic setting. The effect of heat or ice on other important mechanical properties (e.g., reactive stiffness) remains ambiguous and should be the subject of future research, the authors point out. Another systematic review of the literature found limited and inconclusive evidence regarding the effects of CGR on muscle function. The only confirmed positive effect of cryotherapy is the improvement of joint flexibility [47]. These findings show that CC can improve recovery and performance after heavy-weight training. Abaïdia et al. [48], in a study using 20 min CC, showed that it can improve recovery and performance after training with heavy weights.

The dominant view in the scientific literature is that reduced muscle tone and myofascial stiffness affect athletic performance and injury risk. At the same time, the goal is to increase tendon stiffness and muscle elasticity [47], which is to provide optimal conditions for generating muscle strength [49,50]. While less passive muscle stiffness is an indicator of greater flexibility, less active muscle stiffness is associated with a lower force production, as fewer transverse bridges are activated [51]. The relationship between active and passive muscle and tendon stiffness for exercise costs and biomechanics of movement has not been well established, making it difficult to compare the relevant literature [51,52]. Mac Auley [53] indicates, in a systematic review of the literature, that using repeated rather than continuous ice packs helps to maintain reduced muscle temperature without compromising the skin and allows the skin’s surface temperature to return to normal. In contrast, the more profound muscle temperature remains low. Reflexes and motor functions are impaired after permanent CC, as a consequence, patients may be more vulnerable to injury up to 30 min after the procedure. It states that ice is effective but must be applied repeatedly for 10 min. Based on the obtained results, we postulate that 3 min may be sufficient due to the observed changes and the lack of clear dynamics of changes between 3 and 6 min. Alexander et al. [23] observed no significant changes in muscle strength and pain threshold after exposure to 15 min of CC or rest applied immediately after exercise. These authors postulate that multiple performance measurements during warm-up periods within competitive post-workout training schedules are required to develop optimal cooling-to-recovery protocols.

During exercise, plasma diffuses into the intracellular, extracellular, and capillary fluid spaces of the muscles, increasing muscle volume by up to 20% as vascular fluid enters the tissue [54]. Recent findings suggest that the fluid content of muscles may influence the mechanics of force production; however, the extent to which natural volume fluctuations should be expected to influence muscle mechanics in vivo remains unclear. The hypothesis described in the scientific literature is that the increase in passive tension that coincides with the change in volume resulting from the interaction of the intramuscular fluid with the stretchable collagen fibers in the muscle’s extracellular matrix [55]. Compression and cooling can stop or even reverse this process by constricting tiny capillaries and increasing the activity of lymphangion, which is responsible for contractile reactions in the capillary system [56]. Therefore, CC therapy by regulating tissue perfusion may have a beneficial effect on the biomechanical properties of muscles.

### 4.3. Muscle Pain

The bidirectional interaction between expectations and experience can result in self-reinforcing phenomena—so-called “self-fulfilling prophecies”—in many areas of human endeavor, including placebo and nocebo effects in medicine [57]. In pain perception, the self-reinforcement effect has powerful and significant clinical implications. Previous research has shown that expectations of meaning—introduced by prior experience and instruction—cause pain to be regulated to the expected level [58]. When assessing the impact of CGR, the above conclusions should be kept in mind.

In the scientific literature on the analgesic range of CC, the results clearly suggest an analgesic effect. It is observed both in post-traumatic treatment [59] and treatment of chronic neuropathic pain [60]. There are also reports of a beneficial effect on pain and post-exercise changes in different sports discipline groups [23,25,27]. CC used in our study showed an analgesic effect. Turn Mac Auley [53] confirmed the effectiveness of an intermittent, as opposed to continuous, application of cold for pain relief [61]. Moreover, Knobloch et al. [13] also observed a better effect of intermittent cryo-compression using a 10 min stimulation/10 min rest protocol. While CC time studies have yielded significant results, they are still inconclusive.

Nogueira et al. [39], in a systematic review of the literature, showed that topical cryotherapy does not appear to improve delayed onset muscle soreness (DOMS) or exercise-related muscle weakness. The authors pay particular attention to the need for an adequate number of RCTs. Block [62] also found a lack of adequate RCT evidence, but in this case, the authors suggested that there was evidence to support the use of CC to reduce pain and swelling and activate microcirculation. This evidence contradicts observations of the effect of a cold stimulus on the recovery of damaged muscles in animals [63]. Otte et al. [41] and Tomchuk et al. [64] observed that adding pressure to a cold stimulus is more effective than the cold stimulus alone in relieving pain and lowering tissue temperature.

Ostrowski et al. [65] showed that CC might not be sufficient to promote a more significant temperature drop compared to traditional methods such as ice compresses. They observed more significant decreases in traditional methods. A potential reason for this is that the cuff gradually inflates and deflates from 5 to 75 mmHg in the medium setting over a 3 min cycle. Although not explicitly stated in the instruction manual, the premise of this cyclic compression is to mimic muscle contraction, not to help lower the temperature. Contrary to these observations, other authors have found that CC is no different from traditional ice with bandage compression [65,66]. They observed a similar decreases in temperature between the groups. On the other hand, Holwerda et al. [67] observed that applying cold and intermittent pneumatic compression with Game Ready did not cause an acute load on the cardiovascular system over that produced by standard ice packs/flexible compresses. The Game Ready system obtains larger temperature drops at medium- and high-pressure settings. The scientific literature has the most homogeneous results regarding the effect of CC on myalgia. However, at the same time, it should be noted that there is a lack of time estimation data, which did not show statistical significance in our study.

### 4.4. Isometric Muscular Strength

Our research shows that a longer CC time (6 min) does not increase F max, and its value increases significantly in a shorter stimulation time, i.e., 3 min. There is still insufficient evidence for changes in muscle strength with CC [19,23,47,68]. Improvements in maximal strength occur due to a combination of neural and morphological adaptations [69] with the relative contribution of these factors to strength gain with resistance training subject to ongoing debate [70]. Ohnishi and Yamane confirmed the influence of low temperatures on improving the strength and endurance of forearm muscles [68]. The limited available evidence suggests that cold stimuli may attenuate improvements in strength endurance with resistance training, albeit when assessed during single-joint movements involving smaller muscle groups [63]. The physiological mechanisms for the adverse effects of CWI on changes in strength endurance with resistance training remain unclear. The conducted research confirms that cold stimuli influence the hypertrophic reactions of skeletal muscles to the whole body’s resistance training, which directly impacts the amount of measured muscle strength [9]. The results of these studies are mixed, with some suggesting that cold attenuates resistance training-induced increases in muscle cross-sectional area [63]. In contrast, others have found no increase in this parameter after cold application [70,71].

Research on the effects of CC continues to produce many of the conflicting results we mentioned earlier. As Allan et al. [22] reported, we should be aware of important caveats that may arise in certain research situations. Other authors have pointed to the paradox that post-exercise cooling can increase endurance-based muscle adaptability [71] while impairing hypertrophy targets [72].

#### Limitations and Directions of Future Research

The main limitation of these experimental studies was the lack of a control group. The authors are aware of this; however, our assumption was only a preliminary assessment of different durations of CC time. The following results are to be used to evaluate further RCT studies. The study solely includes male MMA fighters, which may restrict the generalizability of the findings to athletes in other sports or to female athletes. Undoubtedly, the group should be increased in subsequent studies, and the time of observation of the changes that occurred should be extended. When planning future research, other factors which could possibly affect personal reactions to CC therapy like nutritional status and hydration level should be considered and investigated before the intervention. Additionally, the influence of changes in stiffness, resting tension, or flexibility in comparison with other methods of post-exercise muscle regeneration and the influence of CC on these parameters in the intervals between efforts may be interesting. Future projects should also focus on assessing and comparing the effects of CC in people with different levels of physical preparation and different sports disciplines and assess the alternation of short-term CC with a break. During the implementation of RCT studies, attention should also be paid to further assessments of CC time parameters. It should also be added that although they objectify the assessment of the impact of CC, the measurement tools used have their previously mentioned limitations. Most of them do not describe reference values. In addition, it should be mentioned that the LDF method is an extremely sensitive method that requires the ability to perform tests with strict procedures that may distort the observed changes. In this case, we should rather use the assessment of changes in response to the applied stimulus (in our case, CC). It should also be noted that for some people, CC therapy is overly favored, and their expectations as to the effect may affect the level of the measured variables. In summary, while the study significantly contributes to understanding CC therapy’s immediate impacts on muscle biomechanics and perfusion in MMA fighters, addressing the mentioned critiques could strengthen future research, enhancing the applicability and understanding of CC therapy’s role in sports recovery.

## 5. Conclusions

The use of cryo-compression therapy in sports still faces many challenges. Our research provides evidence that CC is a stimulus that significantly affects muscle biomechanical changes, pain threshold, strength, and tissue perfusion. It is essential that for the creation of possible protocols, it seems that a 3 min effect is sufficient instead of a 6 min one. Consequently, the effect of intermittent 3 min CC may be worth investigating in future studies.

## Figures and Tables

**Table 1:** Comparisons between the experimental conditions for all measured variables.

Condition	Rest	Post
Perfusion Unit		
CC3	6.67 ± 2.84 (5.34 to 8.00)	5.15 ± 2.34 (4.05 to 6.24)
CC6	7.13 ± 3.21 (5.62 to 8.63)	4.76 ± 1.82 (3.91 to 5.61)
Maximum forearm muscle strength [kgf]		
CC3	46.61 ± 9.18 (42.31 to 50.91)	49.55 ± 8.85 (45.41 to 53.69)
CC6	43.74 ± 8.07 (39.96 to 47.51)	46.20 ± 8.04 (42.44 to 49.96)
Muscle Tone [Hz]		
CC3	18.58 ± 2.06 (17.61 to 19.54)	19.90 ± 2.79 (18.59 to 21.20)
CC6	18.19 ± 1.98 (17.26 to 19.11)	19.66 ± 3.27 (18.13 to 21.19)
Stiffness [N/m]		
CC3	332.5 ± 60.7 (304.1 to 360.9)	366.2 ± 54.4 (340.7 to 391.6)
CC6	325.2 ± 55.0 (299.4 to 350.9)	339.6 ± 53.2 (314.6 to 364.5)
Elasticity [arb]		
CC3	0.95 ± 0.08 (0.92 to 0.99)	1.06 ± 0.17 (0.98 to 1.14)
CC6	0.93 ± 0.12 (0.87 to 0.99)	1.00 ± 0.14 (0.93 to 1.07)
Pressure pain threshold [N/cm]		
CC3	101.1 ± 16.0 (93.6 to 108.6)	134.6 ± 23.3 (123.7 to 145.5)
CC6	94.1 ± 18.0 (85.6 to 102.5)	135.0 ± 21.0 (125.2 to 144.9)

All data are presented as mean ± SD and range.

## Data Availability

Data are available from the corresponding author only at the justified request of a scientist.
